# The epidemiology and microbiological characteristics of infections caused by Gram-negative bacteria in Qatar: national surveillance from the Study for Monitoring of Antimicrobial Resistance Trends (SMART): 2017 to 2019

**DOI:** 10.1093/jacamr/dlad086

**Published:** 2023-08-03

**Authors:** Mazen A Sid Ahmed, Hawabibee Mahir Petkar, Thoraya M Saleh, Mohamed Albirair, Lolita A Arisgado, Faiha K Eltayeb, Manal Mahmoud Hamed, Muna A Al-Maslamani, Abdul Latif Al Khal, Hussam Alsoub, Emad Bashir Ibrahim, Hamad Abdel Hadi

**Affiliations:** Philadelphia Department of Public Health, Laboratory Services, Philadelphia, USA; Division of Microbiology, Department of Laboratory Medicine and Pathology, Hamad Medical Corporation, Doha, Qatar; Division of Microbiology, Department of Laboratory Medicine and Pathology, Hamad Medical Corporation, Doha, Qatar; Department of Global Health, University of Washington, Seattle, USA; Division of Microbiology, Department of Laboratory Medicine and Pathology, Hamad Medical Corporation, Doha, Qatar; Division of Microbiology, Department of Laboratory Medicine and Pathology, Hamad Medical Corporation, Doha, Qatar; Division of Microbiology, Department of Laboratory Medicine and Pathology, Hamad Medical Corporation, Doha, Qatar; Division of Infectious Diseases, Communicable Diseases Centre, Hamad Medical Corporation, Doha, Qatar; Division of Infectious Diseases, Communicable Diseases Centre, Hamad Medical Corporation, Doha, Qatar; Division of Infectious Diseases, Communicable Diseases Centre, Hamad Medical Corporation, Doha, Qatar; Division of Microbiology, Department of Laboratory Medicine and Pathology, Hamad Medical Corporation, Doha, Qatar; Biomedical Research Centre, Qatar University, Doha, Qatar; Division of Infectious Diseases, Communicable Diseases Centre, Hamad Medical Corporation, Doha, Qatar

## Abstract

**Background:**

The global Study of Monitoring Antimicrobial Resistance Trends (SMART) is a surveillance program for evaluation of antimicrobial resistance (AMR) in Gram-negative bacteria (GNB) from different regions including Gulf countries.

**Objectives:**

To evaluate AMR in GNB from various clinical specimens including microbiological and genetic characteristics for existing and novel antimicrobials.

**Methods:**

A prospective study was conducted on clinical specimens from Hamad Medical Corporation, Qatar, between 2017 and 2019 according to the SMART protocol. Consecutive GNB from different sites were evaluated including lower respiratory, urinary tract, intrabdominal and bloodstream infections.

**Results:**

Over the 3 years study period, 748 isolates were evaluated from the specified sites comprising 37 different GNB outlining four key pathogens: *Escherichia coli*, *Klebsiella pneumoniae*, *Pseudomonas aeruginosa* and *Stenotrophomonas maltophilia*.

For the two major pathogens *E. coli* and *K. pneumoniae*, phenotypic ESBL was identified in 55.77% (116/208) compared to 39% (73/187), while meropenem resistance was 3.8% compared to 12.8% and imipenem/relebactam resistance was 2.97% compared to 11.76%, respectively. The overall ceftolozane/tazobactam resistance for *E. coli* was 9.6% (20/208) compared to 14.97% (28/187) for *K. pneumoniae* while resistance for ceftazidime/avibactam was 3.65% (5/137) and 5.98% (10/117), respectively. Genomic characteristics of 70 Enterobacterales including 48 carbapenem-resistant, revealed prevalence of β-lactamases from all classes, predominated by *bla*_CXM-15_ while carbapenem resistance revealed paucity of *bla*_KPC_ and dominance of *bla*_OXA-48_ and *bla*_NDM_ resistance genes.

**Conclusions:**

Surveillance of GNB from Qatar showed prevalence of key pathogens similar to other regions but demonstrated significant resistance patterns to existing and novel antimicrobials with different underlying resistance mechanisms.

## Background

In modern healthcare, challenges of antimicrobial resistance (AMR) have a major impact on public health, with significant morbidity and mortality as well as escalating costs of management.^1^ The consequences of AMR are particularly witnessed in Gram-negative bacteria (GNB) where the pathogens are responsible for a wide spectrum of community and healthcare-associated infections (HAIs), ranging from mild to severe that require critical care and frequently fatal outcomes. Over the last decades, the accumulation of diverse resistance mechanisms in GNB, led to the development of the notorious multidrug resistant organisms (MDROs) with critical consequences.^1,2^ The emergence of MDROs with limited therapeutic alternatives, has been associated with detrimental patient outcomes leading to prolonged length of hospital stay that necessitates urgent prevention and control strategies that include the development of novel antimicrobials options.^3^ Globally GNB encompassing MDROs, are the leading cause of human infections for all age groups, as they are the principal cause of urinary tract infections (UTIs) as well as hospital-acquired respiratory tract infections (RTIs).^4,5^ Similarly, they are amongst the leading causes of nosocomial bacteraemia as well as complicated or uncomplicated intra-abdominal infections (IAIs).^6–9^

When examining the global problem of AMR, it is clear it has regional variations attributed to pathogens and host factors as well as variance in local settings including antimicrobial prescribing choices and consumption, dominance of highly resistant clones as well as variable adherence to infection control and prevention measures.^10^ Furthermore, regional epidemiology does not only differ in prevalence, but also in microbiological characteristics and underlying resistance mechanisms. While extended-spectrum β-lactamases (ESBLs) are the most observed global resistance mechanism in GNB including Enterobacterales, other advanced mechanisms such as those observed in carbapenem-resistant Enterobacterales (CREs) have a different disease spectrum and are predicted to pose significant future challenges.^1^ In CREs, the underlying mechanisms of AMR are diverse, for example, while class A *bla*_KPC_ is the dominant mechanism in North America and Europe, class B such as *bla*_NDM_, *bla*_VIM_ as well as class D *bla*_OXA_ type CREs are dominant in the Middle East and Gulf countries.^11^ Similarly, for *Pseudomonas aeruginosa* and *Acinetobacter baumannii*, resistance mechanisms are dominated by ESBLs and class C cephalosporinases in addition to other antimicrobial permeability resistance mechanisms such as efflux pumps and porins mutations that are unique in the study of the evolution resistance in GNB.^12,13^ Furthermore, antimicrobial characteristics for existing and novel antimicrobials therapy demonstrate regional variations that merit further evaluation to enhance clinical experience as well as aid research and development of future antimicrobial therapy.^14^

The high rate of AMR calls for accurate regional and global surveillance to assess pathogens epidemiology, microbiological and genomic characteristics that remains of paramount importance at all levels particularly for guidance of appropriate antimicrobial therapy. Consequently, in 2015, the World Health Organization developed a global AMR action plan that advocates regional and national monitoring strategies including implementing viable surveillance concepts.^15^

This study is part of the global surveillance of AMR in collaboration with the International Health Management Associates, Inc (IHMA), examining microbiological and genomic characteristics of selected GNB between 2017 and 2019 from Qatar. It includes the evaluation of *in vitro* susceptibility of GNB to the novel antimicrobial agents: imipenem/relebactam, ceftolozane/tazobactam, ceftazidime-avibactam and meropenem-vaborbactam compared to existing comparators from clinical practice, as well as molecular characterization of ESBLs, carbapenemases, plasmid and chromosomally encoded AmpC β-lactamases from specific aerobic Gram-negative species.

## Materials and methods

### Bacterial strains

All isolates were collected from specimens received at the central microbiology laboratory in Qatar according to the criteria in the SMART protocol as outlined. The laboratory receives specimens from 10 general and specialized facilities within Hamad Medical Corporation, which is the main provider of hospital services within the State of Qatar.

Specimens were collected from hospitalized patients from designated facilities. For each year of the study, consecutive clinically relevant isolates of aerobic GNB were collected from patients with lower RTIs (100 isolates), UTIs, (50 isolates) and IAIs (50 isolates) as well as from bloodstream infections (BSIs) (50 isolates). Only one isolate per patient per species was allowed. Isolate demographic information was documented on provided worksheets as per the study protocol. Following local identification using automated BD Phoenix™ Microbiology System (BD Diagnostics, Durham, NC, USA), isolates were transferred to IHMA for further analysis. Identification of all isolates received at each testing facility were confirmed using MALDI-TOF spectrometry (Bruker Daltonics, Billerica, MA, USA). Organism collection, transport, identification and confirmation, quality assurance and centralized database development and management were coordinated by IHMA (Schaumburg, IL, USA). Results were extracted and analysed from the central database by the study reporting group: https://globalsmartsite.com

### Antimicrobials susceptibility testing (ASTs)

Minimum inhibitory concentrations (MICs) were determined at IHMA’s US and European laboratories using frozen in-house custom or commercially available broth microdilution panels. Separate custom panel configurations were made for isolates of Enterobacterales and Gram-negative non-fermenter species (*Acinetobacter* species, *Pseudomonas* species and *Burkholderia* species). All broth microdilution tests were set up according to the Clinical and Laboratory Standards Institute (CLSI) guidelines and MIC interpretive criteria used were those published in 2020 M100 guidelines by CLSI.^16^ Because not all GNB are tested against standard antimicrobial panels as well as some protocol changes during collection period that allowed incremental introduction of novel antimicrobials, not all isolates were uniformly tested against designated antimicrobials, which explains the non-congruent figures. Additionally, interpretive criteria for imipenem/relebactam were those assigned by CLSI, EUCAST and the United States Food and Drug Administration (US FDA).

Using CLSI guidelines, *Escherichia coli*, *Klebsiella pneumoniae*, *K. oxytoca* and *Proteus mirabilis* were classified as ESBL producers if there was at least an 8-fold reduction (i.e. three doubling dilutions) of the MIC for ceftazidime or cefotaxime tested in combination with clavulanic acid versus their MIC values when tested alone.^16^ Quality control (QC) of broth microdilution panels followed the manufacturer’s instructions and CLSI guidelines, using the following ATCC strains: *E. coli* ATCC 25922, *P. aeruginosa* ATCC 27853, *K. pneumoniae* ATCC 700603 and *K. pneumoniae* BAA-2814. Results were included in the analysis only when corresponding QC values tested were within the acceptable ranges as specified by CLSI.

### Molecular characterization of β-lactamase genes

Enterobacterales isolates that met one or more of the following criteria (based on CLSI breakpoints) were screened for the presence of β-lactamase genes: Enterobacterales non-susceptible to ceftolozane/tazobactam (MICs ≥4 mg/L) and non-*Morganellaceae* Enterobacterales, excluding *Serratia* species, non-susceptible to imipenem (MICs ≥2 mg/L) and/or imipenem/relebactam (MICs ≥2 mg/L). *P. aeruginosa* isolates that met one or more of the following criteria were screened for the presence of β-lactamase genes: isolates non-susceptible to ceftolozane/tazobactam (MICs ≥8 mg/L) and isolates non-susceptible to imipenem (MICs ≥4 mg/L) and/or imipenem/relebactam (MICs ≥4 mg/L). The proportion of isolates that met the testing criteria that were characterized was determined based on budgetary constraints which included 70 MDR-GNB and 48 CREs. Qualifying Enterobacterales isolates were screened for the presence of β-lactamase genes (*bla*) encoding class A ESΒLs *bla*_TEM_, *bla*_SHV_, *bla*_CTX-M_, *bla*_VEB_, *bla*_PER_, and *bla*_GES_; *bla*_AmpC_, class B metallo-β-lactamase (MBL) genes *bla*_NDM_, *bla*_IMP_, *bla*_VIM_, *bla*_GIM_ and *bla*_SPM_, class C β-lactamase genes *bla*_ACC_, *bla*_ACT_, *bla*_CMY_, *bla*_DHA_, *bla*_FOX_, *bla*_MIR_ and *bla*_MOX_; class A carbapenemases *bla*_KPC_ and *bla*_GES_ and class D *bla*_OXA-48*-*like_, by multiplex PCR as described previously.^17^

### Data handling and statistical analysis

Summary of statistics were calculated using R software v.4.1.3. The total number of isolates (*n*), MIC50 (mg/L), MIC90 (mg/L) and MIC range (mg/L) were determined for all antimicrobial agents tested. The percentage of susceptibility (%) was calculated according to both CLSI and EUCAST where available. Direct comparisons were made between different interpretive criteria using published breakpoints for each drug.

### Ethical considerations and data management

The study and collaboration were approved by the Medical Research Centre of Hamad Medical Corporation (HMC), which abides by local and international research standards (ref. 17248/17). The study also received approval from the Ethical Committee and Institution Review Board of HMC after demonstrating utmost commitment towards observing outlined standards for data management and sharing including limited access to nominated primary investigators, data anonymity and governance. All shared data with collaborators had no traced patients’ identification.

## Results

### Prevalence and distribution of aerobic GNB isolates

The frequency and distribution of all isolated GNB is shown in Table 1. Thirty-seven different GNB species were isolated, with predominance of four key species accounting for about 80% of infections: *E. coli* (27.9%), *K. pneumoniae* (25%), *P. aeruginosa* (21.9%) and *Stenotrophomonas maltophilia* (6%) while organisms from the order Enterobacterales constituted 67.25% (503/748) of infections. Identified pathogens were isolated from RTIs 39.84% (298/748), IAIs 23.80% (178/748), UTIs at 23.53% (176/748) and BSIs at 12.83% (96/748). *P. aeruginosa* was the most common cause of RTIs and *E. coli* was the most common cause of infections from the three other outlined sites.

**Table 1.** Frequency of Gram-negative organisms collected from blood, intra-abdominal, respiratory and UTIs in the SMART study (2017–2019)

Species	Year of isolation			Site of isolation					Frequency	%
	2017	2018	2019	RT	IA	UT	BS	Unknown		
*Escherichia coli*	72	73	64	8	77	76	48	0	209	27.94%
*Klebsiella pneumoniae*	70	59	58	68	44	55	20	1	187	25%
*Pseudomonas aeruginosa*	53	46	65	108	25	18	13	0	164	21.93%
*Stenotrophomonas maltophilia*	10	22	13	39	5	0	1	0	45	6.02%
*Enterobacter cloacae*	10	9	7	14	7	3	2	0	26	3.48%
*Serratia marcescens*	7	3	10	15	2	2	1	0	20	2.67%
*Acinetobacter baumannii*	8	5	6	15	1	3	1	0	19	2.54%
*Klebsiella aerogenes*	5	4	2	6	1	3	1	0	11	1.47%
*Proteus mirabilis*	4	3	3	1	0	7	2	0	10	1.34%
*Klebsiella variicola*	1	3	4	3	3	0	2	0	8	1.07%
*Enterobacter non-speciated*	2	4	0	5	1	0	0	0	6	0.80%
*Morganella morganii*	2	1	2	0	1	3	1	0	5	0.67%
*Citrobacter freundii*	1	3	0	1	1	1	1	0	4	0.53%
*Salmonella non-speciated*	0	1	3	0	4	0	0	0	4	0.53%
*Acinetobacter non-speciated*	0	2	1	2	0	1	0	0	3	0.40%
*Achromobacter xylosoxidans*	1	1	0	2	0	0	0	0	2	0.27%
*Acinetobacter pittii*	0	1	1	1	1	0	0	0	2	0.27%
*Aeromonas caviae*	1	1	0	0	2	0	0	0	2	0.27%
*Klebsiella oxytoca*	1	1	0	1	1	0	0	0	2	0.27%
*Proteus non-speciated*	0	1	1	1	0	0	1	0	2	0.27%
*Aeromonas hydrophila*	0	1	0	0	1	0	0	0	1	0.13%
*Aeromonas veronii*	0	1	0	0	0	0	1	0	1	0.13%
*Burkholderia cenocepacia*	0	1	0	1	0	0	0	0	1	0.13%
*Burkholderia cepacia*	0	0	1	1	0	0	0	0	1	0.13%
*Burkholderia multivorans*	0	1	0	1	0	0	0	0	1	0.13%
*Citrobacter koseri*	0	0	1	1	0	0	0	0	1	0.13%
*Elizabethkingia miricola*	0	1	0	1	0	0	0	0	1	0.13%
*Enterobacter bugandensis*	0	0	1	1	0	0	0	0	1	0.13%
*Kluyvera ascorbata*	1	0	0	0	1	0	0	0	1	0.13%
*Pantoea non-speciated*	0	0	1	0	0	1	0	0	1	0.13%
*Proteus hauseri*	0	1	0	0	0	0	1	0	1	0.13%
*Proteus penneri*	0	0	1	0	0	1	0	0	1	0.13%
*Providencia stuartii*	0	0	1	0	0	1	0	0	1	0.13%
*Pseudomonas non-speciated*	1	0	0	1	0	0	0	0	1	0.13%
*Pseudomonas otitidis*	0	0	1	1	0	0	0	0	1	0.13%
*Ralstonia non-speciated*	0	1	0	1	0	0	0	0	1	0.13%
*Raoultella ornithinolytica*	0	0	1	0	0	1	0	0	1	0.13%
**Total (%)**	250 (33.42)	250 (33.42)	248^b^ (33.16)	298 (39.84)	178 (23.80)	176 (23.53)	96 (12.83)	1 (0.13)	748 (100)	**100%**

BS, blood stream; IA, intra-abdominal; RT, respiratory tract; UT, urinary tract.

### Demographic profile of study population

The 748 specimens were collected from all age groups (0–99 years), with male preponderance (64.17%). Specimens were from medical, surgical and paediatrics departments while 39.1% of samples were from intensive and critical care units (Supplementary Table S1, available as Supplementary data at *JAC-AMR* Online).

### Antimicrobial susceptibility patterns of GNB isolates

Antimicrobial susceptibility test results for the top 10 species-comprising 699 isolates (93.4%) of the study organisms, are summarized in Table 2.

**Table 2.** Total number and percentages of *in vitro* antimicrobial susceptibility test for common selected Gram-negative organisms collected from blood, intra-abdominal, respiratory and UTIs in the SMART Study (2017–2019)

Species	No. of isolates	AST	Amikacin	Aztreonam	Cefepime	Cefotaxime	Cefoxitin	Ceftazidime	Ceftazidime-avibactam	Ceftolozane-tazobactam	Ceftriaxone	Ciprofloxacin	Colistin	Ertapenem	Imipenem	Imipenem-relebactam	Levofloxacin	Meropenem	Meropenem-vaborbactam	Piperacillin-tazobactam	Tobramycin
*Escherichia coli*	209	No. of tested isolates	209	209	209	72	209	209	137	209	209	145	NA	209	209	209	209	209	64	209	NA
		Susceptible (%)	205 (98.09%)	104 (49.76%)	107 (51.20%)	28 (38.89%)	143 (68.42%)	107 (51.20%)	132 (96.35%)	188 (89.95%)	92 (44.02%)	52 (35.86%)		198 (94.74%)	201 (96.17%)	203 (97.13%)	55 (26.32%)	201 (96.17%)	62 (96.88%)	179 (85.65%)	
*Klebsiella pneumoniae*	187	No. of tested isolates	187	187	187	70	187	187	117	187	187	129	NA	187	187	187	187	187	58	187	NA
		Susceptible (%)	179 (95.72%)	124 (66.31%)	122 (65.24%)	45 (64.29%)	144 (77.01%)	120 (64.17%)	110 (94.02%)	159 (85.03%)	114 (60.96%)	77 (59.69%)		160 (85.56%)	161 (86.10%)	165 (88.24%)	77 (41.18%)	163 (87.17%)	50 (86.21%)	140 (74.87%)	
*Pseudomonas aeruginosa*	164	No. of tested isolates	164	164	164	NA	NA	164	111	164	NA	99	164	NA	164	164	164	164	NA	164	111
		Susceptible (%)	160 (97.56%)	105 (64.02%)	126 (76.83%)			120 (73.17%)	104 (93.69%)	156 (95.12%)		51 (51.52%)	0 (0.00%)		115 (70.12%)	150 (91.46%)	125 (76.22%)	120 (73.17%)		112 (68.29%)	108 (97.30%)
*Stenotrophomonas maltophilia*	45	No. of tested isolates	NA	NA	NA	NA	NA	45	NA	NA	NA	NA	NA	NA	NA	NA	45	NA	NA	NA	NA
		Susceptible (%)						15 (33.33%)									34 (75.56%)				
*Enterobacter cloacae*	26	No. of tested isolates	26	26	26	13	26	26	16	26	26	22	NA	26	26	26	26	26	7	26	NA
		Susceptible (%)	25 (96.15%)	20 (76.92%)	23 (88.46%)	8 (80%)	1 (4.35%)	20 (76.92%)	16 (100%)	20 (76.92%)	19 (27.08%)	16 (84.21%)		23 (88.46%)	23 (88.46%)	24 (92.31%)	16 (61.54%)	24 (92.31%)	7 (100%)	20 (76.92%)	
*Serratia marcescens*	20	No. of tested isolates	20	20	20	7	20	20	13	20	20	10		20	20	20	20	20	10	20	NA
		Susceptible (%)	20 (100%)	20 (100%)	19 (95%)	7 (100%)	1 (5%)	20 (100%)	13 (100%)	19 (95%)	17 (85%)	9 (90%)	NA	19 (95%)	14 (70%)	17 (85%)	11 (55%)	19 (95%)	9 (90%)	18 (90%)	
*Acinetobacter baumannii*	19	No. of tested isolates	19	NA	19	9	NA	19	NA	NA	9	19	19	NA	19	NA	19	19	NA	19	11
		Susceptible (%)	12 (63.16%)		11 (57.89%)	2 (25.00%)		12 (63.16%)			0	8 (61.54%)	0		12 (63.16%)		11 (57.89%)	12 (63.16%)		11 (57.89%)	8 (72.73%)
*Klebsiella aerogenes*	11	No. of tested isolates	11	11	11	5	11	11	6	11	11	9	11	11	11	11	11	11	2	11	NA
		Susceptible (%)	11 (100%)	9 (81.82%)	11 (100%)	3 (60.00%)	0	8 (72.73%)	6 (100%)	10 (90.91%)	8 (72.73%)	6 (66.67%)	11 (100%)	11 (100%)	10 (90.91%)	11 (100%)	5 (45.45%)	11 (100%)	2 (100%)	9 (81.82%)	
*Proteus mirabilis*	10	No. of tested isolates	10	10	10	4	10	10	6	10	10	7	NA	10	10	10	10	10	3	10	NA
		Susceptible (%)	10 (100%)	9 (90%)	9 (90%)	2 (50.00%)	9 (90%)	9 (90%)	6 (100%)	9 (90%)	8 (80%)	5 (71.43%)		10 (100%)	8 (80%)	9 (90%)	5 (50%)	10 (100%)	3 (100%)	10 (100%)	
*Klebsiella variicola*	8	No. of tested isolates	8	8	8	1	8	8	7	8	8	4	NA	8	8	8	8	8	4	8	NA
		Susceptible (%)	8 (100%)	8 (100%)	8 (100%)	1 (100%)	8 (100%)	8 (100%)	7 (100%)	8 (100%)	8 (100%)	4 (100%)		6 (75%)	6 (75%)	6 (75%)	7 (87.5%)	7 (87.5%)	4 (100%)	6 (75%)	

Susceptibility tests were reported according to breakpoints of the CLSI 2020 Edition.

Assessment of the AST of the 503 clinical isolates of Enterobacterales demonstrated that the aminoglycoside amikacin was the most potent, ranging between 95.72% and 100%. *E. coli* demonstrated the highest phenotypic detection of extended-spectrum β-lactamases (ESBLs) at 55.77% (116/208) compared to 39.04% (73/187) for *K. pneumoniae*. Conversely, carbapenem susceptibility patterns of Enterobacterales isolates (*E. coli*, *K. pneumoniae*, *E. cloacae*, *S. marcescens*, *P. mirabilis*, *K. varicola* and *K. aerogenes*) were highly retained being lowest for *K. pneumoniae* (96.17%, 87.17%, 92.31%, 95%, and 100%, respectively). Additionally, further exploration for the new β-lactams/β-lactamase inhibitors (BLBLIs) combinations revealed highest activity for ceftazidime/avibactam with low level resistance (*E. coli*, 3.65%, *K. pneumoniae* 3.98% while for *E. cloacae*, *S. marcescens* and *K. aerogenes* at 0%), respectively.

For the broad BLBLIs, imipenem/relebactam resistance rates were 2.97% for *E. coli* (5/208) compared to 11.76% (22/187) for *K. pneumoniae*. Similarly, for *E*. *coli*, the overall ceftolozane/tazobactam resistance was 9.62% (20/208) compared to 14.97% (28/187) for *K. pneumoniae* while resistance for ceftazidime/avibactam was 3.65% (5/137) and 5.98% (10/117), respectively (Table 2).

Furthermore, assessment of antibacterial activity of 164 *P. aeruginosa* isolates and 19 *A. baumannii* isolates are shown in Table 2. All *P. aeruginosa* isolates showed 100% susceptibility to colistin, with slightly reduced susceptibility to amikacin (97.56%) and tobramycin (97.30%). Unlike other BLBLIs combinations highest susceptibility was observed for ceftolozane/tazobactam (95.12%). All isolates of *A. baumannii* were 100% susceptible to colistin while susceptibility to tobramycin was (72.73%) and (63.16%) for both imipenem and ceftazidime.

### The frequency and distribution of β-lactamase genes among Enterobacterales and P. aeruginosa

Genomic studies of 70 Enterobacterales (32 *K. peumoniae*, 22 *E. coli*, seven *E. cloacae* and nine others), revealed the presence of 157 different β-lactamases resistant genes representing all major classes; class A 71.42% (50/70), class B 24.29% (17/70), class C 25.71% (18/70) and class D 27.14% (19/70) with the overall dominance of the ESBLs *bla*_TEM-OSBL_. Combining microbiological and genetic characteristics, *K. pneumoniae* was more resistant with underlying class B MBLs *bla*_NDM_ and class D OXA-48-like β-lactamases (specifically *bla*_OXA-181_ and *bla*_OXA-232_) when compared to *E. coli* that exhibit mainly class A β-lactamases (Table 3). Additionally, genomic studies of 49 carbapenem-resistant, *P. aeruginosa* isolates, *Pseudomonas*-derived cephalosporinases (PDCs) were the most prevalent resistance genes with predominance of *bla*_PDC-3_ (32.65%, 16/49), *bla*_PDC-19A_ (10.2%, 5/49) and *bla*_PDC-5_ (8.16%, 4/49), while the MBL carbapenemase *bla*_VIM-2_ was detected in only four isolates (8.16%, 4/49), as shown in Table 4.

**Table 3.** The frequency of different β-lactamase genes amongst 70 Enterobacterales isolates collected between 2017 and 2019, which was resistance to different β-lactam antibiotic classes

Organism name	No. of isolates	Class A β-lactamase													Class B β-lactamase							Class C β-lactamase																									Class D β-lactamase				Total
		CTX-M-15	CTX-M-1-240G	CTX-M-TYPE	CTX-M-14	CTX-M-1-TYPE	CTX-M-9-240D	SHV-OSBL	SHV-31	SHV-12	SHV-ESBL	TEM-OSBL	KPC-2	VEB-6	NDM-1	NDM-19	NDM-5	NDM-7	NDM-TYPE	VIM-2	VIM-4	PDC-3	PDC-19A	PDC-5	PDC-1	PDC-12	PDC-14	PDC-37	PDC-35	PDC-39	PDC-TYPE	PDC-11	PDC-117	PDC-15	PDC-16	PDC-59	PDC-60	CMY-2-TYPE	CMY-TYPE	CMY-42	CMY-59	DHA-1	DHA-TRUNC	DHA-TYPE	ACT-TYPE	MIR-TYPE	OXA-181	OXA-232	OXA-48	OXA-TYPE	
*Pseudomonas aeruginosa*	49																			4		16	5	4	3	3	3	3	2	2	2	1	1	1	1	1	1														53
*Klebsiella pneumoniae*	32	16	4	1	1		1	26	3		2	21	1		5		1	3																								1		1			2	13	2		104
*Escherichia coli*	22	7	3	2		1				1		10			1	1	2		2																			4	3	1	1										39
*Enterobacter cloacae*	7														1						1																						1		3	1					7
*Serratia marcescens*	3													1																																				1	2
*Klebsiella variicola*	2																																																2		2
*Klebsiella aerogenes*	2																																									1									1
*Citrobacter freundii*	1																																						1												1
*Proteus mirabilis*	1														1																																				1
Total	119	23	7	3	1	1	1	26	3	1	2	31	1	1	8	1	3	3	2	4	1	16	5	4	3	3	3	3	2	2	2	1	1	1	1	1	1	4	4	1	1	2	1	1	3	1	2	13	4	1	210
Subtotals		101													22							67																									20				210

**Table 4.** The frequency and distribution of different β-lactamase genes among 49 carbapenem-resistant *Pseudomonas aeruginosa* and 48 CREs isolates collect between 2017 and 2019

Species	No. of isolates	Class A β-lactamase											Class B β-lactamase								Class C β-lactamase																			Class D β-lactamase			
		CTX-M-15	CTX-M-1-240G	CTX-M-TYPE	CTX-M-14	CTX-M-1-TYPE	CTX-M-9-240D	SHV-OSBL	SHV-31	SHV-ESBL	TEM-OSBL	KPC-2	NDM-1	NDM-19	NDM-5	NDM-7	NDM-TYPE	VIM-2	VIM-4	PDC-3	PDC-19A	PDC-5	PDC-1	PDC-12	PDC-14	PDC-37	PDC-35	PDC-39	PDC-TYPE	PDC-11	PDC-117	PDC-16	PDC-59	PDC-60	CMY-2-TYPE	CMY-42	DHA-1	DHA-TRUNC	ACT-TYPE	OXA-181	OXA-232	OXA-48	OXA-TYPE	Total no. of genes
*Pseudomonas aeruginosa*	49																	4		16	4	4	2	2	3	3	2	2	2	1	1	1	1	1										49
*Klebsiella pneumoniae*	27	12	4		1	1	1	21	2	2	17	1	5		1	3																					1			1	13	2		88
*Escherichia coli*	11	2	3	1							3		1	1	2		2																		1	1								17
*Enterobacter cloacae*	4												1						1																			1	1					4
*Serratia marcescens*	3																																										1	1
*Klebsiella variicola*	2																																									2		2
*Klebsiella aerogenes*	1																																				1							1
Total	97	14	7	1	1	1	1	21	2	2	20	1	7	1	3	3	2	4	1	16	4	4	2	2	3	3	2	2	2	1	1	1	1	1	1	1	1	1	1	1	13	4	1	161
Subtotals		71									21										50																		19					161

### *Microbiological and genetic characteristics of carbapenem-resistant Enterobacterales and carbapenem-resistant* P. aeruginosa

Microbiological and genetic characteristics of 48 studied CREs (27 *K. pneumonias*, 11 *E. coli*, four *Enterobacter cloacae* and six others) revealed prevalence of ertapenem resistance in 89.58% (43/48), imipenem at 87.5% (42/48) and meropenem at 72.91% (35/48) of clinical isolates with 70.83% (34/48) concordant resistance to all three agents (Table 5). Furthermore, genetic studies revealed extreme rarity of other types of carbapenem-resistance genes with predominance of class D (*bla*_OXA-48_-like type, specifically: 12 *bla_OXA-232,_* 4 *bla*_OXA-48_, 2 *bla*_OXA-181_, and 1 *bla*_OXA-type_) and class B MBL (7 *bla*_NDM-1_, 3 *bla*_NDM-5_, 3 *bla*_NDM-7_, 2 *bla*_NDM-type_, 1 *bla*_NDM-19_ and 1 *bla*_VIM-4_) (Table 4).

**Table 5.** Demographic profile of patients and phenotypic antimicrobial susceptibility among 49 carbapenem-resistant *P. aeruginosa* and 48 CREs isolates collected between 2017 and 2019. Sites of isolation: respiratory tract (RT), intrabdominal (IA), urinary tract UT, blood stream (BS)

Species	*Pseudomonas aeruginosa*	*Klebsiella pneumoniae*	*Escherichia coli*	*Enterobacter cloacae*	*Serratia marcescens*	*Klebsiella variicola*	*Klebsiella aerogenes*	Total (%)
Gender	Number (%)							
Male	37 (75.51)	20 (74.07)	5 (44.45)	1 (25)	3 (100)	2 (100)	1 (100)	69 (71.13)
Female	12 (24.49)	7 (25.93)	6 (54.55)	3 (75)	0	0	0	28 (28.87)
Location								
In-patient	27 (55.1)	21 (77.78)	8 (72.73)	3 (75)	0	0	0	59 (60.82)
Intensive care unit	22 (44.9)	6 (22.22)	3 (27.27)	1 (25)	3 (100)	2 (100)	1 (100)	38 (39.18)
Age								
Paediatric < 14 years	2 (4.08)	1 (3.7)	0	0	0	0	0	3 (3.09)
Adult 14-65 years	31 (63.27)	15 (55.56)	8 (72.73)	3 (75)	3 (100)	2 (100)	1 (100)	63 (64.95)
Geriatric > 65	16 (32.65)	8 (29.63)	3 (27.27)	1 (25)	0	0	0	28 (28.87)
Site of isolation								
RT	36 (73.47)	11 (40.74)	0	1 (25)	3 (100)	1 (50)	1 (100)	53 (54.64)
IA	5 (10.2)	5 (18.52)	6 (54.55)	1 (25)	0	1 (50)	0	18 (18.56)
UT	2 (4.08)	7 (25.93)	5 (45.45)	2 (50)	0	0	0	16 (16.49)
BS	6 (12.24)	4 (14.81)	0	0	0	0	0	10 (10.31)
No. of isolates susceptible to								
Aztreonam	14 (28.57)	9 (33.33)	0	1 (25)	3 (100)	2 (100)	1 (100)	
Cefepime	28 (57.14)	5 (18.52)	0	2 (50)	2 (66.67)	2 (100)	1 (100)	
Cefotaxime	NA	10 (37.04)	0	2 (50)	3 (100)	2 (100)	1 (100)	
Ceftazidime	24 (48.98)	6 (22.22)	0	1 (25)	3 (100)	2 (100)	0	
Ceftriaxone	NA	5 (18.52)	0	1 (25)	2 (66.67)	2 (100)	0	
Colistin	49 (100)	NA	NA	NA	NA	NA	NA	
Meropenem	8 (16.33)	4 (14.81)	3 (27.27)	2 (50)	2 (66.67)	1 (50)	1 (100)	
Ertapenem	NA	1 (3.7)	0	1 (25)	2 (66.67)	0	1 (100)	
Imipenem	0	2 (7.41)	3 (27.27)	1 (25)	0	0	0	
Ceftolozane/tazobactam	41 (83.67)	5 (18.52)	1 (9.09)	1 (25)	2 (66.67)	2 (100)	1 (100)	
Imipenem/relebactam	35 (71.43)	6 (22.22)	5 (45.45)	2 (50)	0	0	1 (100)	
Pipracillin/tazobactam	19 (38.78)	2 (7.41)	0	1 (25)	2 (66.67)	0	1 (100)	
Total (%)	49 (100)	27 (100)	11 (100)	4 (100)	3 (100)	2 (100)	1 (100)	97 (100)

Microbiological evaluation of the 49 carbapenems-resistant *P. aeruginosa* isolates against imipenem and imipenem/relebactam, revealed that 14 isolates were resistant to both (concordant resistance), while 35 *P. aeruginosa* isolates were resistant to imipenem but susceptible to imipenem/relebactam (discordant resistant group) indicating relebactam restored 71.4% (35/49) of imipenem activity. Four of the concordant resistant isolates harboured class B MBL *bla*_VIM2_ while the rest were AmpC-type β-lactamases in the form of PDCs speared by *bla*_PDC-3_. Conversely, the discordant group although showed embedded PDCs including *bla*_PDC-3_ (eight isolates), were of diverse types including *bla*_PDC-19A_, *bla*_PDC-1_, *bla*_PDC-5_, *bla*_PDC-14_ and *bla*_PDC-37_ (five then three isolates each respectively). Furthermore, the eight *P. aeruginosa* isolates that were resistant to ceftolozane/tazobactam harboured the following β-lactamase genes: four *bla*_VIM-2_, four *bla*_PDC-3_, two blaPDC_-5_ and two *bla*_PDC-35_ Supplementary Table S2).

## Discussion

The impact of AMR is a major global threat to humanity because of direct clinical as well as indirect economic and social consequences.^12^ To overcome AMR challenges, the widely adopted recommendation is to implement cornerstone concepts of basic and advanced surveillance studies to assess pathogens evolving microbiological characteristics as well as examine dynamic resistance mechanisms.^18^ Furthermore, following the implementation of conventional practices in modern healthcare such as regular bacterial phenotypic analysis, genotypic and molecular epidemiology has been advocated as crucial advanced concept to face unexpected challenges particularly at different regional healthcare settings.^19^

The SMART is an international research collaboration for the study of AMR in GNB focusing on four key infections: RTIs and UTIs together with IAIs and BSIs. The study started about two decades ago on a small scale then expanded as an ongoing global surveillance study.^17^ In the Middle East and Africa regions, 24 medical centres participated in the study detailed as follows (arranged alphabetically): Israel, Jordan, Kenya, Kuwait, Lebanon, Morocco, Qatar, Saudi Arabia, South Africa, Tunisia and the United Arab Emirates.

The results of surveillance of GNB from secondary and tertiary healthcare from Qatar with emphasis on the four specified sites of infections demonstrated dominance of four key pathogens namely, *E coli*, *K. pneumoniae*, *P. aeruginosa* and *S. maltophiila*, which are in line with published regional surveillance studies.^6,20–23^ Of note, *E. coli* remains the main pathogen for UTIs in contrast to *K. pneuminae* that were isolated mainly from RTIs followed by UTIs and IAIs but with established higher AMR (Table 2). This reflects that *K. pneumoniae* isolates from HAIs are mainly secondary to hospital or ventilation-associated pneumonia, which probably explains the high observed resistance rates. Similar epidemiological studies highlighted escalating MDR-GNB at critical care settings particularly rising trends of extremely-drug resistant *K. pneumoniae.*^21,24,25^

The presented results are the first comprehensive surveillance study in the country, and it reflects an alarmingly high-level AMR profile since for *E. coli*, the prevalence of phenotypic resistance pattern for ESBLs was higher than half of isolates (55.7%) while for *K. pneumoniae* was 39%. Almost a decade earlier, limited studies from Qatar at the existed but smaller healthcare settings focusing on 452 episodes of BSIs, established almost half ESBL prevalence (27.8% for *E. coli* and 17.9% for *K. pneumoniae* respectively*)* while a surveillance study of 629 consecutive Enterobacterales from critical care settings between 2012 and 2013 revealed the overall prevalence of 17.3%.^26,27^

In the Middle East region, focusing on GNB, the escalating problem of AMR is predominated by ESBL production with multifactorial explanations mainly from existing diverse population, frequent influx of seasonal international travellers together with the widely practised inappropriate and high antimicrobial consumption.^28–30^

Although *E. coli* exhibited higher ESBLs phenotypic resistance patterns compared to *K. pneumoniae*, detailed microbiological and genetic characteristics points towards the opposite where *E. coli* demonstrated lower-level resistance to carbapenems when compared to *K. pneumoniae* (meropenem resistance of 3.83% and 12.83%, respectively), which has not changed even for novel agents not currently available at the hospital formulary such as imipenem/relebactam (2.83% and 11.76%, respectively). Similarly, regarding newer agents of BLBLIs such as ceftazidime/avibactam and ceftolozane/tazobactam, in *E. coli* the overall resistance rates for the two agents were 3.65% and 10.05%, while for *K. pneumoniae* these were 5.98% and 14.97%, respectively. Again, this is showing rising trends for the two agents since between 2012 and 2013, when 109 ESBL producing Enterobacterales isolated from critical care were tested against ceftazidime/avibactam and ceftolozane/tazobactam, it demonstrated AMR rates of 0.9%.^31^

Distinctively when 49 carbapenem-resistant *P. aeruginosa* were tested against imipenem and imipenem/relebactam, all isolates were resistant to imipenem. Whereas relebactam restored *in vitro* imipemen activity in 71.4% (35/49) of isolates. Four of these isolates resistant to imipenem/relebactam harboured the MBL *bla*_VIM-2_ while the rest harboured different class C AmpC-type β-lactamases in the form of PDCs. Intriguingly, none of the resistant isolates harboured *bla*_IMP_ as observed elsewhere.^32^ Comparatively, avibactam, which is a potent BLBLI capable of inhibiting class A, C and D β-lactamases but is overwhelmed by class B MBL such as *bla*_NDM_ and *bla*_VIM_ whereas the closely related relebactam has similar inhibition spectrum albeit with absent activity against class D OXA-type carbapenemases, demonstrated supplementary antimicrobial potency.^33^ Of note, for *P. aeruginosa* the classic pearl of wisdom that genotypic resistance patterns does not always equate phenotypic ones because there are other complex resistance mechanisms involving diverse membrane pathways such as the loss of porin channels and overproduction efflux pumps.^34^ While imipenem is more resistant to GNB ejecting efflux pumps when compared to meropenem, it remains susceptible to porin channel mutations that hinder its inward penetration conferring phenotypic resistance.^33,35^ In *P. aeruginosa*, the loss of OprD porin channels together with class C ESBLs and AmpC such as PDC is the hallmark of imipenem resistance.^36^

Among carbapenem-resistant *P. aeruginosa*, eight isolates that were resistant to ceftolozane/tazobactam harboured different β-lactamase genes: class B *bla*_VIM-2_; class C *bla*_PDC-3_, *bla*_PDC-5_ and *bla*_PDC-35_ and class B *bla*_VIM-2_ and *bla*_PDC-35_, which have been associated with high-level resistance to ceftolozane/tazobactam.^37,38^ When comparing discordant results of the *in vitro* activity for the novel BLBLIs for most Enterobacterales, it favours ceftazidime/avibactam over ceftolozane/tazobactam but not for *P. aeruginosa* where the latter demonstrated superior activity. Nevertheless, antimicrobials activity cannot be reliably extrapolated to clinical practice since evaluation for the two agents, demonstrated similar efficacy with no noticeable significant clinical differences.^31,39–41^

Genetic characterization of 70 Enterobacterales including 48 isolates that were CREs revealed the presence of all major β-lactamase classes with a predominance of *bla*_CXM-15_ ESBLs in conjunction with other historic resistant genes such as *bla*_TEM_ and *bla*_SHV_ distributed in *K. pneumoniae* and *E. coli* when compared to other GNB (Tables 3 and 4). The plasmid-mediated ESBL gene, *bla*_CXM-15_ has a global distribution with a direct link to advanced cephalosporins resistance being the most widely reported resistant gene from all global regions including the Middle East and Gulf countries.^27,28,42,43^ Noticeably, *E. coli* resistant genes were mainly class A ESBLs while *K. pneumonia* demonstrated more divergent pattern with the presence of a multitude of class D OXA-type ESBLs as well as carbapenemases such as *bla*_NMD_ and *bla*_OXA-48_ and its closely related *bla*_OXA-181_ and *bla*_OXA-232_. These mutated resistant genes are derivatives from their parent carbapenemase *bla*_OXA-48_ with few point mutations.^44^ Locally, our PCR-based molecular techniques will report these different resistant genes grouped as *bla*_OXA-48_. Distinctively, among the 70 Enterobacterales and 48 CREs only a single *K. pneumoniae* isolate harboured *bla*_KPC-2_, which was probably imported as shown in similar local CREs studies.^45^ The plasmid-mediated KPCs serine carbapenemases are historically linked to the West, particularly North America and Southern Europe, although they have been reported in some other distant countries such as Israel and China but this has been extremely rare in our region.^42,45,46^ Furthermore, current molecular epidemiology, affirms reported and observed dominance of the carbapenemase *bla*_OXA_ types and *bla*_NDM_ in the region.^28,45,47^

Despite the diverse microbiological and genomic outcomes of the study, there are some noticeable limitations. The prospective study collected representative pathogens from specific infection sites that have changed over the study period, which might lessen the overall epidemiological accuracy. Furthermore, for microbiological and genetic testing, although the defined protocol was followed, it did change over time. For example, as novel antibiotics were introduced into clinical practice, they were evaluated but against fewer isolates. Therefore, a true comparison cannot be accurately reported. Last, the methods for genetic and molecular characterization of resistance followed the central study protocol, which is more detailed when compared to local practice. That might generate more elaborative results that are difficult to benchmark at local levels.

In conclusion, the SMART surveillance study from Qatar between 2017 and 2019 encompassed a sizeable collection of 748 isolates comprising 37 different GNB dominated by *E. coli*, *K. pneumoniae*, *P. aeruginosa* and *S. maltophilia* showing significant microbiological and genetic characteristics with a prevalence of divergent types of ARGs particularly *bla*_CXM-15_ whereas *K. pneumonia* isolates collected mainly from respiratory specimens were more resistant to existing as well as novel antimicrobials with a distinct overall dominance of *bla*_OXA_ type and *bla*_NDM_ carbapenemases.

## Supplementary Material

dlad086_Supplementary_Data

## Supplementary data

Tables S1 and S2 are available as Supplementary data at *JAC-AMR* Online.
