# Supplementary material for: The epidemiology and microbiological characteristics of infections caused by Gram-negative bacteria in Qatar: national surveillance from the Study for Monitoring of Antimicrobial Resistance Trends (SMART): 2017 to 2019
Source: JAC Antimicrob Resist. 2023 Aug 3;5(4):dlad086. doi: 10.1093/jacamr/dlad086 (PMC10400155; doi:10.1093/jacamr/dlad086)
Supplement: dlad086_Supplementary_Data [file dlad086_supplementary_data.docx]

**Supplementary Table S1.** Demographic profile of patients with aerobic GNB infections

| **Age group** | **Frequency** | **%** | **Location** | **Frequency** | **%** |
| --- | --- | --- | --- | --- | --- |
| 0 - 9 | 72 | 9.63% | Medicine General | 231 | 30.88% |
| 10 - 19 | 13 | 1.74% | Medicine ICU | 123 | 16.44% |
| 20 - 29 | 48 | 6.42% | Emergency Room | 105 | 14.04% |
| 30 - 39 | 78 | 10.43% | Surgery General | 84 | 11.23% |
| 40 - 49 | 114 | 15.24% | Surgery ICU | 77 | 10.29% |
| 50 - 59 | 113 | 15.11% | General Unspecified ICU | 53 | 7.09% |
| 60 - 69 | 122 | 16.31% | Pediatric ICU | 40 | 5.35% |
| 70 - 79 | 95 | 12.70% | None Given | 27 | 3.61% |
| 80 - 89 | 85 | 11.36% | Pediatric General | 8 | 1.07% |
| 90-99 | 8 | 1.07% | **Gender** |  |  |
|  |  |  | Female | 268 | 35.83% |
|  |  |  | Male | 480 | 64.17% |
| **Total** | 748 | 100.00% | **Total** | 748 | 100.00% |

Respiratory tract specimens include sputum, broncho-alveolar lavage, endotracheal aspirate lavage, others. Intra-abdominal specimens include abscesses, appendix, colon, gallbladder, liver, pancreas, peritoneal fluid, small intestine, stomach and others.

ICU, intensive care unit

**Supplementary Table S2:** Demographic characteristics, molecular characterization, and susceptibility testing results for 119 β-lactamase producing GNB isolates tested, collected from between 2017 and 2019.

| **Organism Name** | **Age** | **Gender** | **Site of Isolation** | **Aztreonam** | **Cefepime** | **Cefotaxime** | **Cefoxitin** | **Ceftazidime** | **Ceftriaxone** | **Ertapenem** | **Imipenem** | **Meropenem** | **Pipracillin/tazobactam** | **Ceftolozane/tazobactam** | **Imipenem/relebactam** | **Meropenem/vaborbactam** | **ESBL Test** | **Molecular β-lactamase gene profile** |
| --- | --- | --- | --- | --- | --- | --- | --- | --- | --- | --- | --- | --- | --- | --- | --- | --- | --- | --- |
| *Pseudomonas aeruginosa* | 94 | Male | RTI: Endotracheal aspirate | R | R | NA | NA | R | NA | NA | R | R | R | S | S | NA | NEG | PDC-1; |
| *Pseudomonas aeruginosa* | 56 | Male | RTI: Endotracheal aspirate | S | S | NA | NA | R | NA | NA | R | R | S | S | S | NA | NEG | PDC-1; |
| *Pseudomonas aeruginosa* | 76 | Male | RTI: Sputum | S | S | S | NA | S | NA | NA | R | S | S | S | S | NA | NEG | PDC-1; |
| *Pseudomonas aeruginosa* | 68 | Male | RTI: Endotracheal aspirate | R | S | NA | NA | S | NA | NA | R | R | R | S | S | NA | NEG | PDC-11; |
| *Pseudomonas aeruginosa* | 3 | Male | CVS: Blood | R | R | NA | NA | R | NA | NA | R | R | R | S | R | NA | NEG | PDC-117; |
| *Pseudomonas aeruginosa* | 56 | Male | RTI: Other | R | R | NA | NA | R | NA | NA | R | R | R | S | S | NA | NEG | PDC-12; |
| *Pseudomonas aeruginosa* | 14 | Female | RTI: Sputum | R | S | NA | NA | R | NA | NA | R | R | R | S | S | NA | NEG | PDC-12; |
| *Pseudomonas aeruginosa* | 41 | Male | CVS: Blood | S | S | NA | NA | S | NA | NA | R | S | S | S | S | NA | NEG | PDC-12; |
| *Pseudomonas aeruginosa* | 60 | Male | RTI: Sputum | R | S | NA | NA | S | NA | NA | R | R | R | S | S | NA | NEG | PDC-14; |
| *Pseudomonas aeruginosa* | 51 | Male | RTI: Sputum | R | S | NA | NA | S | NA | NA | R | R | R | S | S | NA | NEG | PDC-14; |
| *Pseudomonas aeruginosa* | 58 | Male | RTI: Endotracheal aspirate | R | S | NA | NA | S | NA | NA | R | R | S | S | S | NA | NEG | PDC-14; |
| *Pseudomonas aeruginosa* | 28 | Male | UTI: Urine | R | S | NA | NA | S | NA | NA | R | S | S | S | S | NA | NEG | PDC-15; |
| *Pseudomonas aeruginosa* | 55 | Male | RTI: Sputum | R | S | NA | NA | S | NA | NA | R | R | S | S | S | NA | NEG | PDC-16; |
| *Pseudomonas aeruginosa* | 87 | Male | RTI: Sputum | S | S | NA | NA | S | NA | NA | R | R | R | S | S | NA | NEG | PDC-19A; |
| *Pseudomonas aeruginosa* | 87 | Male | RTI: Endotracheal aspirate | S | S | NA | NA | S | NA | NA | R | R | S | S | S | NA | NEG | PDC-19A; |
| *Pseudomonas aeruginosa* | 51 | Male | RTI: Sputum | S | S | NA | NA | S | NA | NA | R | S | S | S | S | NA | NEG | PDC-19A; |
| *Pseudomonas aeruginosa* | 54 | Male | RTI: Endotracheal aspirate | S | S | NA | NA | S | NA | NA | R | S | S | S | S | NA | NEG | PDC-19A; |
| *Pseudomonas aeruginosa* | 34 | Male | RTI: Sputum | S | S | NA | NA | S | NA | NA | R | S | S | S | S | NA | NEG | PDC-19A; |
| *Pseudomonas aeruginosa* | 81 | Female | RTI: Endotracheal aspirate | R | S | NA | NA | R | NA | NA | R | R | R | S | S | NA | NEG | PDC-3; |
| *Pseudomonas aeruginosa* | 86 | Male | RTI: Endotracheal aspirate | R | R | NA | NA | R | NA | NA | R | R | R | S | S | NA | NEG | PDC-3; |
| *Pseudomonas aeruginosa* | 23 | Female | RTI: Endotracheal aspirate | R | R | NA | NA | R | NA | NA | R | R | R | S | S | NA | NEG | PDC-3; |
| *Pseudomonas aeruginosa* | 81 | Female | RTI: Sputum | R | S | NA | NA | R | NA | NA | R | R | R | S | R | NA | NEG | PDC-3; |
| *Pseudomonas aeruginosa* | 22 | Female | RTI: Endotracheal aspirate | R | R | NA | NA | R | NA | NA | R | R | R | S | R | NA | NEG | PDC-3; |
| *Pseudomonas aeruginosa* | 42 | Male | RTI: Sputum | R | R | NA | NA | R | NA | NA | R | R | R | S | R | NA | NEG | PDC-3; |
| *Pseudomonas aeruginosa* | 72 | Female | CVS: Blood | R | R | NA | NA | R | NA | NA | R | R | R | R | R | NA | NEG | PDC-3; |
| *Pseudomonas aeruginosa* | 49 | Male | IAI: Pancreas | R | R | NA | NA | R | NA | NA | R | R | R | R | R | NA | NEG | PDC-3; |
| *Pseudomonas aeruginosa* | 53 | Female | RTI: Sputum | R | R | NA | NA | R | NA | NA | R | R | R | S | S | NA | NEG | PDC-3; |
| *Pseudomonas aeruginosa* | 81 | Male | RTI: Endotracheal aspirate | R | R | NA | NA | R | NA | NA | R | R | R | S | S | NA | NEG | PDC-3; |
| *Pseudomonas aeruginosa* | 50 | Male | IAI: Pancreas | S | S | NA | NA | S | NA | NA | R | R | S | S | S | NA | NEG | PDC-3; |
| *Pseudomonas aeruginosa* | 43 | Male | RTI: Endotracheal aspirate | S | S | NA | NA | S | NA | NA | R | R | S | S | S | NA | NEG | PDC-3; |
| *Pseudomonas aeruginosa* | 19 | Male | UTI: Urine | R | S | NA | NA | S | NA | NA | R | R | S | S | S | NA | NEG | PDC-3; |
| *Pseudomonas aeruginosa* | 49 | Male | CVS: Blood | R | S | NA | NA | S | NA | NA | R | R | S | S | R | NA | NEG | PDC-3; |
| *Pseudomonas aeruginosa* | 82 | Male | RTI: Sputum | S | R | NA | NA | R | NA | NA | R | R | R | R | R | NA | NEG | PDC-3; VIM-2; |
| *Pseudomonas aeruginosa* | 80 | Male | RTI: Endotracheal aspirate | S | R | NA | NA | R | NA | NA | R | R | R | R | R | NA | NEG | PDC-3; VIM-2; |
| *Pseudomonas aeruginosa* | 41 | Male | CVS: Blood | R | R | NA | NA | R | NA | NA | R | R | R | R | R | NA | NEG | PDC-35; VIM-2; |
| *Pseudomonas aeruginosa* | 50 | Male | RTI: Sputum | R | R | NA | NA | R | NA | NA | R | R | R | R | R | NA | NEG | PDC-35; VIM-2; |
| *Pseudomonas aeruginosa* | 82 | Male | RTI: Endotracheal aspirate | R | R | NA | NA | R | NA | NA | R | R | R | S | S | NA | NEG | PDC-37; |
| *Pseudomonas aeruginosa* | 78 | Male | RTI: Endotracheal aspirate | R | S | NA | NA | S | NA | NA | R | R | S | S | S | NA | NEG | PDC-37; |
| *Pseudomonas aeruginosa* | 58 | Female | RTI: Endotracheal aspirate | S | S | NA | NA | S | NA | NA | R | S | S | S | S | NA | NEG | PDC-37; |
| *Pseudomonas aeruginosa* | 60 | Female | IAI: Other | R | R | NA | NA | R | NA | NA | R | R | R | S | S | NA | NEG | PDC-39; |
| *Pseudomonas aeruginosa* | 46 | Male | IAI: Gall Bladder | R | S | NA | NA | S | NA | NA | R | R | R | S | R | NA | NEG | PDC-39; |
| *Pseudomonas aeruginosa* | 64 | Female | RTI: Endotracheal aspirate | R | R | NA | NA | R | NA | NA | R | R | R | R | S | NA | NEG | PDC-5; |
| *Pseudomonas aeruginosa* | 49 | Female | RTI: Endotracheal aspirate | R | R | NA | NA | R | NA | NA | R | R | R | R | R | NA | NEG | PDC-5; |
| *Pseudomonas aeruginosa* | 53 | Male | IAI: Pancreas | R | S | NA | NA | S | NA | NA | R | R | S | S | S | NA | NEG | PDC-5; |
| *Pseudomonas aeruginosa* | 66 | Male | CVS: Blood | S | S | S | NA | S | NA | NA | R | S | S | S | S | NA | NEG | PDC-5; |
| *Pseudomonas aeruginosa* | 65 | Female | RTI: Sputum | R | R | NA | NA | R | NA | NA | R | R | R | S | S | NA | NEG | PDC-59; |
| *Pseudomonas aeruginosa* | 37 | Male | RTI: Sputum | R | S | NA | NA | S | NA | NA | R | R | R | S | R | NA | NEG | PDC-60; |
| *Pseudomonas aeruginosa* | 53 | Male | RTI: Sputum | R | R | NA | NA | R | NA | NA | R | R | R | S | S | NA | NEG | PDC-TYPE; |
| *Pseudomonas aeruginosa* | 53 | Male | RTI: Endotracheal aspirate | R | S | NA | NA | S | NA | NA | R | R | S | S | S | NA | NEG | PDC-TYPE; |
| *Klebsiella pneumoniae* | 46 | Male | RTI: Sputum | R | S | S | R | R | R | S | S | S | R | R | S | S | NEG | DHA-TYPE; |
| *Klebsiella pneumoniae* | 78 | Male | RTI: Sputum | R | R | R | S | R | R | S | S | S | R | R | S | NA | POS | SHV-31; TEM-OSBL; CTX-M-15; |
| *Klebsiella pneumoniae* | 42 | Male | UTI: Urine | R | R | R | R | R | R | R | R | R | R | R | R | NA | POS | SHV-31; TEM-OSBL; CTX-M-15; OXA-232 |
| *Klebsiella pneumoniae* | 89 | Female | UTI: Urinary Bladder | R | R | R | R | R | R | R | R | R | R | R | R | NA | POS | SHV-31; TEM-OSBL; CTX-M-15; OXA-232 |
| *Klebsiella pneumoniae* | 49 | Female | RTI: Sputum | R | R | R | R | R | R | R | R | R | R | R | R | NA | POS | SHV-ESBL(e); TEM-OSBL(b); CTX-M-15; OXA-232(c) |
| *Klebsiella pneumoniae* | 81 | Male | IAI: Other | R | R | R | R | R | R | R | R | R | R | R | R | R | POS | SHV-ESBL; TEM-OSBL; CTX-M-1-240G; OXA-232 |
| *Klebsiella pneumoniae* | 79 | Female | UTI: Urine | R | R | R | S | S | R | R | R | R | R | R | R | NA | POS | SHV-OSBL(b); CTX-M-TYPE; OXA-48(c) |
| *Klebsiella pneumoniae* | 68 | Female | RTI: Sputum | S | S | S | R | S | S | R | R | S | R | S | R | NA | NEG | SHV-OSBL(b); OXA-181(c) |
| *Klebsiella pneumoniae* | 72 | Male | UTI: Urine | S | S | S | S | S | S | R | S | S | R | S | S | NA | NEG | SHV-OSBL(b); OXA-181(c) |
| *Klebsiella pneumoniae* | 23 | Male | IAI: Other | R | R | R | R | R | R | R | R | R | R | R | S | NA | POS | SHV-OSBL(b); TEM-OSBL(b); CTX-M-14; KPC-2; |
| *Klebsiella pneumoniae* | 67 | Male | UTI: Urine | R | R | R | R | R | R | S | S | S | R | R | S | NA | POS | SHV-OSBL(b); TEM-OSBL(b); CTX-M-15; |
| *Klebsiella pneumoniae* | 81 | Female | UTI: Urine | R | R | R | R | R | R | S | S | S | S | R | S | NA | POS | SHV-OSBL(b); TEM-OSBL(b); CTX-M-15; |
| *Klebsiella pneumoniae* | 1 | Male | RTI: Endotracheal aspirate | R | R | R | R | R | R | R | R | R | R | R | R | NA | POS | SHV-OSBL(b); TEM-OSBL(b); CTX-M-15; NDM-1; |
| *Klebsiella pneumoniae* | 43 | Male | RTI: Endotracheal aspirate | R | R | R | R | R | R | R | R | R | R | R | R | NA | POS | SHV-OSBL(b); TEM-OSBL(b); CTX-M-15; OXA-232(c) |
| *Klebsiella pneumoniae* | 58 | Male | IAI: Gall Bladder | R | R | R | R | R | R | R | R | R | R | R | R | NA | POS | SHV-OSBL(b); TEM-OSBL(b); CTX-M-15; OXA-48(c) |
| *Klebsiella pneumoniae* | 48 | Male | RTI: Endotracheal aspirate | S | S | S | R | S | S | R | R | R | R | S | R | NA | NEG | SHV-OSBL; |
| *Klebsiella pneumoniae* | 37 | Male | RTI: Sputum | S | S | S | S | S | S | S | R | S | S | S | R | S | NEG | SHV-OSBL; |
| *Klebsiella pneumoniae* | 82 | Male | IAI: Abscess | S | S | S | R | R | S | R | R | S | S | S | S | NA | NEG | SHV-OSBL; DHA-1; |
| *Klebsiella pneumoniae* | 74 | Female | CVS: Blood | S | R | R | R | R | R | R | R | R | R | R | R | R | NEG | SHV-OSBL; NDM-7; |
| *Klebsiella pneumoniae* | 83 | Male | CVS: Blood | S | R | R | R | R | R | R | R | R | R | R | R | R | NEG | SHV-OSBL; NDM-7; |
| *Klebsiella pneumoniae* | 73 | Male | CVS: Blood | S | R | S | R | S | R | R | R | R | R | R | R | R | NEG | SHV-OSBL; OXA-232 |
| *Klebsiella pneumoniae* | 42 | Male | RTI: Sputum | S | R | S | R | S | R | R | R | R | R | R | R | NA | NEG | SHV-OSBL; OXA-232 |
| *Klebsiella pneumoniae* | 74 | Female | CVS: Blood | R | R | R | R | R | R | R | R | R | R | R | R | R | POS | SHV-OSBL; TEM-OSBL; CTX-M-1-240G; CTX-M-9-240D; NDM-1; |
| *Klebsiella pneumoniae* | 65 | Male | RTI: Sputum | R | R | R | R | R | R | R | R | R | R | R | R | R | POS | SHV-OSBL; TEM-OSBL; CTX-M-1-240G; NDM-1; OXA-232 |
| *Klebsiella pneumoniae* | 52 | Male | UTI: Urine | R | R | R | R | R | R | R | R | R | R | R | R | R | POS | SHV-OSBL; TEM-OSBL; CTX-M-1-240G; NDM-5; |
| *Klebsiella pneumoniae* | 73 | Female | RTI: Sputum | R | R | S | S | R | R | S | S | S | R | R | S | NA | POS | SHV-OSBL; TEM-OSBL; CTX-M-15; |
| *Klebsiella pneumoniae* | 58 | Female | UTI: Urinary Bladder | R | R | R | R | R | R | R | R | R | R | R | R | NA | POS | SHV-OSBL; TEM-OSBL; CTX-M-15; NDM-1; |
| *Klebsiella pneumoniae* | 58 | Male | UTI: Urine | R | R | R | R | R | R | R | R | R | R | R | R | NA | POS | SHV-OSBL; TEM-OSBL; CTX-M-15; NDM-1; OXA-232 |
| *Klebsiella pneumoniae* | 58 | Male | RTI: Endotracheal aspirate | R | R | S | R | R | R | R | R | R | R | R | S | NA | POS | SHV-OSBL; TEM-OSBL; CTX-M-15; OXA-232 |
| *Klebsiella pneumoniae* | 64 | Male | RTI: Endotracheal aspirate | R | R | S | S | R | R | R | R | R | R | R | S | NA | POS | SHV-OSBL; TEM-OSBL; CTX-M-15; OXA-232 |
| *Klebsiella pneumoniae* | 64 | Male | RTI: Endotracheal aspirate | R | R | S | S | R | R | R | S | R | R | R | S | NA | POS | SHV-OSBL; TEM-OSBL; CTX-M-15; OXA-232 |
| *Klebsiella pneumoniae* | 38 | Male | IAI: Gall Bladder | R | R | R | R | R | R | R | R | R | R | R | R | NA | POS | TEM-OSBL(b); CTX-M-15; NDM-7; |
| *Escherichia coli* | 45 | Female | IAI: Other | R | R | R | R | R | R | R | R | R | R | R | R | NA | NEG | CMY-42; NDM-TYPE; |
| *Escherichia coli* | 52 | Female | IAI: Stomach | R | R | R | R | R | R | R | S | S | R | R | S | NA | NEG | CMY-TYPE; |
| *Escherichia coli* | 61 | Female | UTI: Urine | R | NA | S | R | R | R | S | S | S | R | R | S | NA | NEG | CMY-TYPE; |
| *Escherichia coli* | 38 | Male | IAI: Abscess | R | R | R | R | R | R | R | R | R | R | R | S | S | POS | CTX-M-1-240G; |
| *Escherichia coli* | 53 | Male | IAI: Pancreas | R | R | R | R | R | R | R | R | R | R | R | S | S | POS | CTX-M-1-240G; |
| *Escherichia coli* | 58 | Female | UTI: Urine | R | R | R | R | R | R | R | R | R | R | R | R | R | POS | CTX-M-1-240G; NDM-1; |
| *Escherichia coli* | 68 | Female | UTI: Urine | R | R | R | R | R | R | R | S | S | R | R | S | NA | POS | CTX-M-15; |
| *Escherichia coli* | 78 | Female | BS | R | NA | S | S | S | R | S | S | S | S | R | S | NA | POS | CTX-M-15; |
| *Escherichia coli* | 45 | Female | IAI: Other | R | R | R | S | R | R | S | S | S | S | R | S | NA | POS | CTX-M-15; |
| *Escherichia coli* | 39 | Male | bloodstream | R | R | R | R | R | R | S | S | S | R | R | S | S | POS | CTX-M-1-TYPE; CMY-2-TYPE; |
| *Escherichia coli* | 57 | Male | IAI: Peritoneal Fluid | R | R | R | R | R | R | R | R | R | R | R | R | NA | POS | CTX-M-TYPE; NDM-TYPE; |
| *Escherichia coli* | 70 | Male | RTI: Bronchoalveolar lavage | S | S | S | R | S | S | S | S | S | S | R | S | S | NEG | NA |
| *Escherichia coli* | 9 | Male | UTI: Urine | R | R | R | S | R | R | S | S | S | R | R | S | NA | POS | SHV-12(e); TEM-OSBL(b); |
| *Escherichia coli* | 78 | Female | UTI: Urine | R | R | R | R | R | R | R | S | S | R | S | S | NA | POS | TEM-OSBL(b); CTX-M-15; |
| *Escherichia coli* | 32 | Male | IAI: Abscess | R | R | R | R | R | R | R | R | R | R | R | R | NA | POS | TEM-OSBL(b); CTX-M-15; NDM-19; |
| *Escherichia coli* | 45 | Male | UTI: Urine | R | R | R | R | R | R | R | R | R | R | R | R | NA | POS | TEM-OSBL(b); CTX-M-15; NDM-5; |
| *Escherichia coli* | 33 | Female | IAI: Peritoneal Fluid | R | R | R | R | R | R | S | S | S | R | R | S | S | NEG | TEM-OSBL; CMY-2-TYPE; |
| *Escherichia coli* | 33 | Female | IAI: Pancreas | R | R | R | R | R | R | S | S | S | R | R | S | S | NEG | TEM-OSBL; CMY-2-TYPE; |
| *Escherichia coli* | 84 | Female | UTI: Urine | R | R | R | R | R | R | R | R | R | R | R | R | R | NEG | TEM-OSBL; CMY-2-TYPE; NDM-5; |
| *Escherichia coli* | 41 | Male | IAI: Abscess | R | NA | S | R | R | R | S | S | S | R | R | S | NA | NEG | TEM-OSBL; CMY-TYPE; |
| *Escherichia coli* | 66 | Male | UTI: Urine | R | R | S | R | R | R | S | S | S | R | R | S | NA | POS | TEM-OSBL; CTX-M-15; CMY-59; |
| *Escherichia coli* | 33 | Female | IAI: Peritoneal Fluid | R | R | R | R | R | R | S | S | S | R | R | S | NA | POS | TEM-OSBL; CTX-M-TYPE; |
| *Enterobacter cloacae* | 51 | Male | RT | R | NA | S | R | R | R | S | S | S | R | R | S | S | NEG | ACT-TYPE; |
| *Enterobacter cloacae* | 60 | Male | intra-abdominal | R | S | S | R | R | R | S | S | S | R | R | S | S | NEG | ACT-TYPE; |
| *Enterobacter cloacae* | 60 | Female | IAI: Abscess | R | R | R | R | R | R | R | R | R | R | R | R | NA | NEG | ACT-TYPE; VIM-4; |
| *Enterobacter cloacae* | 31 | Male | urinary tract | R | R | R | R | R | R | R | R | R | R | R | R | NA | NEG | DHA-TRUNC; NDM-1; |
| *Enterobacter cloacae* | 77 | Female | RTI: Endotracheal aspirate | S | S | S | R | S | S | S | R | S | S | S | S | S | NEG | MIR-TYPE; |
| *Enterobacter cloacae* | 63 | Female | UTI: Urinary Bladder | R | S | S | R | R | R | R | S | S | R | R | S | NA | NEG | NA |
| *Enterobacter cloacae* | 40 | Male | RTI: Sputum | R | S | S | R | R | R | S | S | S | R | R | S | NA | NEG | NA |
| *Serratia marcescens* | 25 | Male | RTI: Sputum | S | S | S | R | S | S | S | R | S | S | S | R | NA | NEG | NA |
| *Serratia marcescens* | 60 | Male | RTI: Sputum | S | S | S | R | S | S | S | R | S | S | S | R | NA | NEG | NA |
| *Serratia marcescens* | 50 | Male | RTI: Sputum | S | R | S | R | S | R | R | R | R | R | R | R | R | NEG | OXA-TYPE |
| *Klebsiella aerogenes* | 26 | Male | RTI: Sputum | S | S | S | R | R | R | S | R | S | S | S | S | NA | NEG | DHA-1; |
| *Klebsiella aerogenes* | 59 | Male | RTI: Sputum | R | S | R | R | R | R | S | S | S | R | R | S | NA | NEG | NA |
| *Klebsiella variicola* | 50 | Male | RTI: Sputum | S | S | S | S | S | S | R | R | R | R | S | R | S | NEG | OXA-48 |
| *Klebsiella variicola* | 50 | Male | IAI: Peritoneal Fluid | S | S | S | S | S | S | R | R | S | R | S | R | S | NEG | OXA-48 |
| *Citrobacter freundii* | 66 | Male | respiratory tract | R | R | S | R | R | R | S | S | S | R | R | S | NA | NEG | CMY-TYPE; |
| *Proteus mirabilis* | 68 | Male | UTI: Urine | R | R | R | S | R | R | S | S | S | S | R | S | NA | POS | VEB-6; |

IAIs, intra-abdominal infections; RTIs, respiratory tract infections; UTIs, urinary tract infections

R; resistance, S, susceptible, NA, not available
